# Healthcare Costs Following Medical Gender‐Affirmation: Evidence From Whole‐of‐Population Australian Administrative Data

**DOI:** 10.1002/hec.70127

**Published:** 2026-07-05

**Authors:** Karinna Saxby, Dennis Petrie, Brendan J. Nolan

**Affiliations:** ^1^ The Melbourne Institute of Applied Economic and Social Research The University of Melbourne Melbourne Australia; ^2^ Centre for Health Economics Monash Business School Caulfield Australia; ^3^ Trans Health Research Group University of Melbourne Melbourne Victoria Australia; ^4^ Department of Endocrinology Austin Health Melbourne Australia; ^5^ Equinox Gender Diverse Clinic Thorne Harbour Health Abbotsford Victoria Australia

**Keywords:** Australia, gender‐affirming care, healthcare costs, nonbinary, out‐of‐pocket costs, transgender

## Abstract

Gender incongruence in trans and nonbinary (“trans”) populations is often associated with psychological distress and increased demand for mental healthcare. Gender‐affirming hormone therapy (GAHT) is a key component of care for many trans people, yet long‐term evidence around its cost implications remains limited despite increasing uptake and policy attention worldwide. We provide the first population‐based evidence on the healthcare costs of GAHT initiation using longitudinal administrative data from 32,313 trans Australians who initiated testosterone‐based GAHT (tGAHT) or estradiol‐based GAHT (eGAHT) between 2013 and 2024. Employing a dynamic difference‐in‐differences design, we estimate the impacts on government expenditure and patient out‐of‐pocket costs in the 6 years after initiation, using future initiators as controls. We find that initiating tGAHT leads to an additional AUD$3119 (USD$2246) in government expenditure and AUD$143 (USD$103) in out‐of‐pocket costs over 6 years; corresponding estimates were AUD$8348 (USD$6011) and AUD$1269 (USD$914) for eGAHT recipients. For tGAHT recipients, both government and out‐of‐pocket costs declined after initiation, with mental healthcare reductions exceeding ongoing hormone therapy expenditure after 6 years. For eGAHT recipients, costs also fell but remained above baseline, driven by sustained prescription spending. Taken together with quality‐of‐life benefits, our results suggest GAHT is likely to be cost‐effective.

## 1 Introduction

People identifying as transgender and nonbinary (“trans”) represent a growing proportion of the population, with global estimates ranging from 0.3% to 0.5% among adults and 1.2%–2.7% among children and adolescents (Zhang et al. 2020). Some but not all trans people experience gender dysphoria, which refers to the distress individuals experience when their gender identity does not align with their sex recorded at birth (Dhejne et al. 2016). Gender dysphoria has been linked to significant healthcare costs, particularly in relation to mental health treatment (S. J. Kim et al. 2024; Grochtdreis et al. 2025). While mental healthcare is likely critical in preventing some severe outcomes such as self‐harm or suicide, for many trans people it may function as a temporary or supportive measure rather than addressing underlying causes of gender dysphoria (Ludwig et al. 2009; Saxby, Buchmueller, et al. 2025; Bower et al. 2011; L. F. Campbell et al. 2013; Dhejne et al. 2016). Evidence suggests that medical gender‐affirmation—which can be used to support alignment between physical characteristics and individuals' gender identity (Cheung et al. 2019)—may provide a more effective and long‐term treatment option for many trans people (Poteat et al. 2023).

This can involve puberty suppression (for young people), gender‐affirming hormone therapy (GAHT), and gender‐affirming surgical interventions. GAHT is the most commonly accessed type of medical gender‐affirmation, with between 70% and 80% of trans people wanting to, or already accessing, GAHT (K. Baker and Restar 2022; Cook et al. 2017).

Access, public subsidization, and, indeed, legalization of gender‐affirming medical care varies significantly across different jurisdictions (H.‐H. Kim et al. 2025; Turban et al. 2021). Indeed, recently many countries have implemented bans on access to gender‐affirming medical care, with some decision makers citing uncertainty around the long‐term benefits, potential harms, and costs (Queensland Government 2025; Barbee and Rosen 2025; Kennedy 2025; Noone et al. 2025).

Rigorous evidence on the benefits, risks, and long‐term cost implications of gender‐affirming medical care is essential to guide regulatory and funding decisions across jurisdictions, and to enable individuals to make informed treatment choices. In addition, in a universal healthcare system such as Australia's, evaluating the incremental benefits of gender‐affirming medical care, in terms of health outcomes and net economic impacts, is critical for informing public subsidization decisions.

Universal health systems also aim to ensure equitable access to clinically effective care, while stewarding public resources and maintaining system sustainability. This is particularly salient given evidence that trans populations experience substantial health disparities extending beyond mental health conditions, including higher all‐cause and cause‐specific mortality relative to cisgender populations (Saxby, Bailey, et al. 2026). In this context, decisions regarding access and subsidization may have implications not only for efficiency, but also for reducing inequities in health outcomes.

To date the evidence base has largely focused on changes in mental health following GAHT initiation, finding that GAHT is associated with short‐term improvements in quality of life, reductions in gender dysphoria, and decreased depression, anxiety, and suicidal ideation (Nolan et al. 2023; K. E. Baker et al. 2021; Mann et al. 2024; T. Campbell et al. 2023). Other studies have documented the direct payer and patient costs of gender‐affirming healthcare itself (Downing et al. 2022; K. Baker and Restar 2022; Chu et al. 2025; Das and Dusetzina 2023) or modeled the potential cost‐effectiveness of insurance coverage of gender‐affirming healthcare using abstracted outcomes and standardised unit costs (Padula et al. 2016).

To our knowledge, no studies have investigated the long‐term health system costs following initiation of medical gender‐affirmation—an evidence gap which is particularly notable given that gender‐affirming medical care is likely to be a partial substitute for long‐term mental healthcare (Wright et al. 2023; Bretherton et al. 2021; Saxby and Nolan 2025) and extensive policy debates around its subsidization and even legalization (Australian Government 2025; Mann and Barbee 2025). Here, we contribute to the evidence base by providing the first population‐level estimates of how GAHT initiation affects both government and patient out‐of‐pocket healthcare costs in a universal healthcare setting. Drawing on longitudinal administrative data for 32,313 trans Australians who initiated testosterone‐based GAHT (tGAHT) or estradiol‐based GAHT (eGAHT) between 2013 and 2024, we applied a dynamic difference‐in‐differences framework to estimate within‐individual changes in costs 6 years after initiation, using future initiators as controls. Analyses were stratified by regimen (tGAHT or eGAHT). Given that GAHT has been shown to improve mental health among trans people, we further disaggregated our results by type of healthcare, distinguishing ongoing hormone therapy expenditure from changes in out‐of‐hospital mental healthcare costs.

We find that GAHT initiation is associated with dynamic and heterogeneous changes in healthcare costs, peaking in the initiation year before declining substantially. Total average government costs over 6 years are AUD$3119 (USD$2246) for tGAHT recipients and AUD$8348 (USD$6011) for eGAHT recipients, with total additional patient out‐of‐pocket costs of AUD$143 (USD$103) and AUD$1269 (USD$914) respectively.^1^ For tGAHT recipients, government spending toward hormone therapy is more than offset by reductions in mental healthcare costs. For eGAHT recipients, costs also declined but remained above pre‐initiation levels across the follow‐up period. Taken together with other evidence of the quality of life and mental health impacts estimated in earlier studies (Nolan et al. 2023; Manieri et al. 2014; Tordoff et al. 2022; T. Campbell et al. 2023), suggests that both regimens are likely to be cost‐effective under typical willingness‐to‐pay thresholds.^2^ Moreover, if the observed declining trends in healthcare costs continue in the future, along with sustained or improved mental health benefits,^3^ then the value for money of GAHT will be even higher.

This study provides several unique contributions. To our knowledge, we provide the first population‐based empirical evidence that initiation of GAHT has distinct and dynamic cost implications for both governments and patients in a universal healthcare setting. In particular, the persistence of higher costs among eGAHT recipients highlights the importance of considering regimen‐specific trajectories when evaluating these economic impacts.

These results also carry clear policy implications in the context of mounting legislative bans on gender‐affirming care worldwide (Queensland Government 2025; Barbee and Rosen 2025). Combined with evidence that GAHT, on average, substantially improves mental health and quality of life among trans people (Nolan et al. 2023; Manieri et al. 2014; T. Campbell et al. 2023), our finding that long‐term healthcare expenditure can return to, or even fall below, pre‐initiation levels suggests that not only, is GAHT likely to be a cost‐effective intervention, but may lead to broader savings in the healthcare system. Restricting access to this care will likely impose long‐term health and economic effects for patients and healthcare payers, particularly when considering the mental health burden and associated healthcare costs of not providing treatment.

The remainder of this paper is set out as follows. Section 2 provides an overview of the institutional background of Australia's universal health insurance program and access to medical gender‐affirmation. Section 3 describes the potential cost implications associated with GAHT initiation. Sections 4 and 5 respectively describe the data and empirical strategy. Section 6 presents the results of the analyses. Sections 7 and 8 conclude by summarizing the key findings, situating them in relation to earlier research, and outlining the main policy implications.

## 2 Institutional Setting

All Australian citizens and permanent residents are entitled to free or subsidized healthcare under Australia's universal health insurance scheme, Medicare. Medicare provides free services in public hospitals and subsidizes out‐of‐hospital medical services and prescription medicines to varying degrees (Krassnitzer 2020).

Medical practitioners can set their own fees for services listed on the Medicare Benefits Schedule (MBS) and these fees are not regulated. If the provider charges the same fee as the benefit paid by the government (i.e., equal to 100%, 85% or 75% of the set schedule fee pending the service type), this service is said to be “bulk billed” and the patient incurs no out‐of‐pocket cost (Department of Health 2020). If service providers charge a fee above the benefit paid (or indeed, charges the MBS schedule fee or higher for non‐General Practitioner (GP) services) the individual pays the gap between the schedule fee and the benefit paid. On top of the benefit paid, GPs are provided with additional financial incentives to bulk bill concession card holders (low income groups, welfare beneficiaries, and pensioners) (Kecmanovic and Hall 2015; Van Der Weyden 2004).

Subsidized prescription medicines are available under the Pharmaceutical Benefits Scheme (PBS). In the community, all individuals pay co‐payments for PBS‐funded prescription medicines, with lower fees for concessional patients. In 2024, the prescription medicine co‐payment was AUD7.70 for concessional patients and AUD31.60 for general patients. The majority of prescription medicines in Australia are accessed through the PBS.

Mental health services are also subsidized under Medicare, including consultations with GPs, psychiatrists, and psychologists. As trans populations experience elevated rates of psychological distress and mental health conditions relative to the general population, engagement with subsidized mental health services is common, with recent evidence indicating that trans people use on average 3–7 times more Medicare mental health services than the general population in Australia (Saxby, Buchmueller, et al. 2026; AIHW 2025; Saxby, Hutchinson‐Tovar, et al. 2025). In addition, trans Australians experience broader health disparities, including higher all‐cause and cause‐specific mortality relative to cisgender populations (Saxby, Bailey, et al. 2026; Wooden et al. 2025).

One of the core aims of Medicare and Australia's broader National Medicines Policy is to ensure that all Australians have “equitable access to healthcare” (Department of Health and Aged Care 2024; Department of Health and Aged Care 2022) and allocate resources according to clinical need while maintaining sustainability. Against this backdrop, evaluating the health and economic consequences of GAHT is important both for addressing inequities and for considering the opportunity costs associated with public subsidization. The health system cost implications of GAHT are particularly relevant as GAHT may reduce psychological distress and partially substitute for ongoing mental health service utilisation.

As in many other countries, gender‐affirming healthcare in Australia is typically categorized into three stages: puberty suppression (Stage 1), hormone therapy (Stage 2), and surgical intervention (Stage 3). Access and funding arrangements vary markedly across these stages, reflecting a complex mix of public and private provision and variation in state and federal responsibilities. Puberty blockers are used to delay the onset or progression of puberty in adolescents. These medications suppress the production of sex hormones (testosterone or estrogen) and in most jurisdictions, these are issued through publicly funded gender clinics. However, outside these pathways, access often depends on private prescriptions, with patients bearing the full out‐of‐pocket costs. This creates inequities in access based on geographic proximity to specialist services and ability to pay.

GAHT is subsidized under the PBS, although patients still incur a co‐payment. GAHT for individuals assigned female at birth usually involves injectable or transdermal testosterone, while GAHT for individuals assigned male at birth includes oral or transdermal estradiol, typically alongside an anti‐androgen (Hembree et al. 2017; Cheung et al. 2019). While availability under the PBS improves affordability, there remain barriers to access: not all GPs are willing to prescribe GAHT, and access often depends on referral to specialists. For individuals treated with tGAHT, PBS subsidy requires consultation with, or a second opinion from, a specialist such as an endocrinologist, sexual health physician, or urologist (AusPATH 2022).

Most people undertake GAHT before surgery. Surgeries that may be part of gender‐affirmation for transgender people include genital surgeries (including phalloplasty or vaginoplasty; gonadectomy); chest surgeries (e.g., mastectomy or mammoplasty); and facial surgeries (K. Baker and Restar 2022). Gender‐affirming surgeries are available in the public system in a small number of jurisdictions but there is generally limited service availability and long waiting times. As there is no consistent Medicare funding pathway for many surgical procedures, most patients access surgery privately (including internationally) and pay out‐of‐pocket.

These institutional features shape both the distribution of costs across sectors and the potential fiscal implications of GAHT within Australia's universal healthcare system.

## 3 Potential Health Cost Implications of Medically Transitioning

There are likely to be multifaceted and dynamic costs associated with initiation of GAHT. Clinical guidelines dictate that expenditure in the first year will be in part attributable to GP visits, mental healthcare, endocrinologist visits, and monitoring, such as blood tests, that usually becomes less frequent after stabilization (Cundill 2020). Clinical guidelines recommend frequent monitoring following initiation of GAHT, typically every 3 months for the first year before reducing to one or two appointments per annum (Hembree et al. 2017). As gender‐affirmation is a highly individualized process, in the first years after initiation, individuals may titrate GAHT levels up or down to achieve their desired effects. It is also likely that over time some individuals may reduce their GAHT levels. Taken together, we expect that both individual and government healthcare costs may spike in the first years around initiation and reduce thereafter.

There are also likely to be distinct differences by regimen. For example, the masculinizing effects of tGAHT are generally quite rapid, achieving more noticeable physical changes such as deepened voice and increased muscle mass, which can be profoundly affirming. Conversely, the feminizing effects of estrogen, such as breast development and fat redistribution, are much slower and can take many years to reach their full effect. Moreover, for people assigned male at birth who have already experienced male puberty, irreversible changes such as voice deepening and bone structure will not be altered by GAHT (Hembree et al. 2017). These clinical differences in efficacy are also thought to contribute, in part, to some of the larger improvements in emotional wellbeing observed following initiation in tGAHT recipients compared to eGAHT recipients (L. Foster Skewis et al. 2021).

The social realities of medical transition can also differ for more “masculinizing” or “feminizing” gender‐affirmation pathways. For example, social systems that favor masculinity over femininity have been proposed as an explanation for why trans feminine people face greater discrimination in employment and subsequent economic stressors relative to trans masculine people (Hughto et al. 2015). Consistent with this, research investigating transitions (based on gender marker and gender‐congruent first name changes or medical gender‐affirmation) find larger post‐transition earnings penalties among trans feminine people (Carpenter et al. 2024; de Weerd et al. 2024). Altogether, if social stressors are larger and physical changes accrue more slowly for eGAHT recipients, improvements in mental health and related reductions in healthcare costs may arrive later and be less pronounced than for tGAHT recipients.

Finally, trans people face structural, cultural, and administrative barriers that depress baseline healthcare engagement (Hughto et al. 2015) and to this end, GAHT initiation may serve as a “gateway” to address other unmet needs (immunizations, cancer screening, cardiometabolic risk). Other studies have also noted that trans people assigned male at birth use less healthcare than trans people assigned female at birth (Saxby, Buchmueller, et al. 2026) which may raise expenditure initially among eGAHT recipients before longer‐run offsets emerge.

## 4 Data

The data for this analysis comes from the Person Level Integrated Data Asset (PLIDA), an individual‐level linked dataset which combines information from population Census and various administrative data sources including healthcare records, social security records, income, and death records (ABS 2022). Healthcare records capture publicly subsidized out‐of‐hospital healthcare services and prescription medications for all Australian citizens and permanent residents (provided under Medicare). As below prescription medication co‐payment was not available prior to April 2012, we sourced data from April 2012 to September 2024 (the most recent and complete data available at the time of writing).

The prescription medication records contain information on an individual's “gender marker.” Historically, this marker, which is recorded as “male” or “female” (and therefore does not capture non‐binary or other diverse gender identities) is categorized based on sex at birth. Individuals have been able to update their gender marker with Medicare since 2013, however currently only changes to “male” or “female” are provided in the prescription medicines data (Services Australia 2024).

Following the previous literature (Nolan et al. 2024; Saxby and Nolan 2025), we identified trans people accessing GAHT using a combination of information on gender markers and prescription medication use. Namely, trans people accessing tGAHT were classified as those who ever used testosterone and had a current or previous female gender marker while trans people accessing eGAHT were classified as those who ever used estradiol and had a current or previous male gender marker between 2012 and 2024. To increase confidence that we were observing an individual's initiation of GAHT, we excluded all individuals who were already receiving GAHT in 2012 (i.e., the earliest year available in our Medicare records).

Finally, following previous studies, we excluded individuals who initiated at under 15 years and individuals who used alternating estradiol and testosterone treatment^4^ (Gülgöz et al. 2019; K. Baker and Restar 2022; Glintborg et al. 2023; de Blok et al. 2021; Saxby and Nolan 2025). As outcomes were constructed at the annual level (calendar year), the analytic window spans 2013 to 2024. All individuals were observed from age 12 years until death or the end of the observation window (30 September 2024), whichever occurred first. Healthcare costs contributed prior to age 12 was not included.

The descriptive statistics for the study sample are presented in Table 1. Between 2013 and 2024, 11,868 individuals initiated tGAHT and 20,445 initiated eGAHT. The median age at initiation was lower for tGAHT recipients (25 years) than for eGAHT recipients (34 years). 65% of tGAHT initiators were between 15 and 25 years at initiation, compared with 56% of eGAHT initiators.

**TABLE 1:** Descriptive statistics.

	Testosterone‐based GAHT (*n* = 11,868) Mean/prop.	Estradiol‐based GAHT (20,445) Mean/prop.
Age first start GAHT	24.96	34.00
Year start GAHT		
2013	0.01	0.05
2014	0.01	0.05
2015	0.02	0.05
2016	0.03	0.06
2017	0.05	0.05
2018	0.08	0.06
2019	0.09	0.07
2020	0.09	0.08
2021	0.12	0.10
2022	0.14	0.13
2023	0.17	0.14
2024	0.18	0.15
Age group start GAHT		
15–24 years	0.65	0.44
≥ 25 years	0.35	0.56
No. years observed post GAHT	4.08	4.85
Annual government expenditure (before GAHT)		
Out‐of‐hospital services and prescription medicines	1578.21	1528.22
Out‐of‐hospital services	1247.93	955.09
Prescription medicines	330.28	573.12
Annual out‐of‐pocket costs (before GAHT)		
Out‐of‐hospital services and prescription medicines	333.98	289.64
Out‐of‐hospital services	223.17	161.88
Prescription medicines	110.81	127.76

*Note:* All costs presented in 2024 AUD.

The distribution of treatment initiation has also shifted over time. In 2013–2014, 93% of GAHT initiators accessed eGAHT, but by 2023–2024, tGAHT accounted for ∼41% of initiations. As such, the average follow‐up was larger for eGAHT initiators (4.9 years) relative to tGAHT initiators (4.1 years).

Although the eGAHT sample was older relative than tGAHT sample, in the years prior to initiation, eGAHT recipients had lower levels of annual government healthcare expenditure than tGAHT recipients (AUD$1528 vs. AUD$1578) and incurred lower out‐of‐pocket costs (AUD$290 vs. AUD$334). This aligns with other research indicating that among trans people, those assigned male at birth have lower baseline utilization than those assigned female at birth (Saxby, Buchmueller, et al. 2026).

## 5 Empirical Strategy

To empirically estimate the impact of GAHT initiation on healthcare costs, we analyze healthcare costs between 2013 and 2024 and exploit the variation in the time in which individuals first initiate GAHT within this period. This approach is particularly relevant in this context as one would expect that differences in healthcare costs between trans and cisgender individuals to vary substantially in terms of both the level differences and the trends in utilization over time.

The key identification assumption for this approach is the parallel trends assumption: that outcomes for trans people initiating GAHT earlier would have, in the absence of GAHT, evolved in parallel to trans people who initiate GAHT later. In addition to visually assessing statistically significant differences through overlapping confidence intervals in an event study approach (Maghsoodloo and Huang 2010), we test the joint nullity of pre‐treatment trends.

Our baseline specification follows the approach by De Chaisemartin and d'Haultfoeuille (2020) to estimate dynamic treatment effects with staggered treatment adoption as follows:

(1) $$Y_{it}=\alpha +\sum_{k=-7,k\neq -3}^{5}\beta_{k}D_{k,it}+\mu_{i}+\theta_{it}+\tau_{y}+\in_{it}$$

where Y denotes healthcare costs (government expenditure, patient out‐of‐pocket) for individual i within period t, $D_{k}$ are event time indicators (leads and lags) for time relative to GAHT initiation^5^ ($k=t-G_{i}$ , where $G_{i}$ is the GAHT initiation period for individual i), $\mu_{i}$ are individual fixed effects (which accounts for all observed and unobserved time‐invariant heterogeneity), $\theta_{it}$ are time‐varying age bands (12–17, 18–24, 25–29, …, 60–64, 65 years plus), and $\in_{it}$ is the error term. To account for secular changes that may have impacted healthcare use and prescribing over time, we included a full set of calendar year fixed effects in $\tau_{y}$. This captures both linear and nonlinear time trends common to all individuals (e.g., secular changes that could impact mental health or access to healthcare over time, such as national policy changes or broader economic conditions). Standard errors are clustered at the individual level, the level at which the treatment occurs.

This empirical approach has several strengths. While the previous literature has use cisgender individuals as comparators, as aforementioned, it is unlikely that outcomes between trans and cisgender people evolve in parallel over time. This approach also ensures that we avoid biases associated with using already treated individuals as controls (Callaway and Sant’Anna 2021, De Chaisemartin and d'Haultfoeuille 2020).

The event study was estimated separately by regimen type (i.e., tGAHT or eGAHT) for 7 years before and 6 years after GAHT initiation, with all event‐time coefficients referenced to 3 years before GAHT initiation (i.e., event time =−3). We selected year “–3” (rather than “–2” or “–1” which are more commonly used) as the baseline period to minimize bias from anticipatory changes in healthcare use and costs in the years immediately preceding GAHT initiation. In practice, access to GAHT often involves a multi‐year pathway of engagement with providers, referrals, assessments, and related care, which may begin well before the first prescription is dispensed. As a result, healthcare costs may already begin to shift in the immediate pre‐initiation period, meaning that years “–1” and “–2’ may not represent a stable “pre‐treatment” counterfactual. This choice also echoes similar analyses in this space which acknowledges that gender‐affirmation is a multi‐year process (Carpenter et al. 2024; Saxby, Buchmueller, et al. 2026; Saxby and Nolan 2026).

We then conduct several robustness checks. First, given the distributional features of cost data, we apply alternate functional forms (Mullahy and Norton 2024). We first transform outcomes (i.e., ln*(costs+0.01)*) and then adopt a two‐part modeling approach where we estimate a linear probability model on the probability of incurring positive costs and then, conditional on positive expenditure, estimate the effects on spending levels. This approach allows us to separately identify extensive‐ and intensive‐margin responses to GAHT initiation. While this framework addresses distributional features of cost data, it does not resolve selection into GAHT, which reflects barriers to access and unmet need; accordingly, our estimates should be interpreted as effects among trans people who access GAHT.

The research design assumes that the timing of GAHT initiation is effectively random, conditional on fixed effects. This assumption is likely reasonable when comparing individuals who begin treatment around the same time but may be less credible for comparisons between those whose initiation dates differ by many years. People who delay treatment, for instance, might differ in important ways such as health status, socioeconomic status, or access to healthcare. In particular, the latter may have been influenced by policy changes over the study period such as expansions to the number of subsidized psychologist sessions, physician incentives, and COVID‐19 telehealth arrangements.

A comparison of age at initiation and outcomes (government spending and out‐of‐pocket expenditure) in the reference period suggests that there are important level differences in outcomes (Supporting Information S1: SM.1). For example, people who initiated GAHT in later years are younger than those who initiated earlier. While level differences in government spending seem similar across cohorts of earlier and later initiators, out‐of‐pocket costs for later GAHT initiators appear to be somewhat higher. However this pattern may also reflect nationwide trends in increasing out‐of‐pocket costs (Saxby and Zhang 2025).

Given these differences, we conduct two additional analyses. First, we explore how the effects varied for individuals who initiated GAHT between 15 and 24 years or between 25 years and above. Specifically, we chose these bands to align with the WHO definition of trans youth (World Health Organization 2025).

Second, for each “treated” cohort, we repeat the main analysis by restricting the control group to be people who initiated GAHT within 3 years. This analysis requires several sample restrictions because our healthcare data spans 2013 to 2024, that is, individuals who initiate between 2013 and 2016 will not equally contribute to each event time coefficient. For example, for someone who initiates in 2016, their first observed event time in calendar year 2013 is “–3” and no pre‐reference periods are observed. To conduct this robustness check, we therefore restrict the sample to those who initiate between 2017 and 2021 to investigate the shorter‐run effects on costs in the year of initiation (i.e., event time =0). That is, someone who initiates in 2017 will only be compared to those who initiated in 2018, 2019, and 2020 while someone who initiates in 2021 is compared to those who initiate in 2022, 2023, and 2024. While this approach mechanically reduces the follow‐up time, we can compare the costs in the immediate years following initiation to costs in the baseline model. If the results are similar, this will provide some reassurance that effects are not driven by cohort differences in initiation years.

Following the main estimation and robustness checks, we examine out‐of‐hospital health service costs and prescription medication costs. We then further breakdown the cost categories into GAHT‐related costs (testosterone, estradiol, and anti‐androgens), mental healthcare costs (mental health services, antidepressants, and anxiolytics), and GP visits. All costs were inflated into 2024 using the Consumer Price Index (ABS 2024). All analyses were conducted using STATA version 18.

## 6 Results

### 6.1 Event Study Results

**FIGURE 1:**
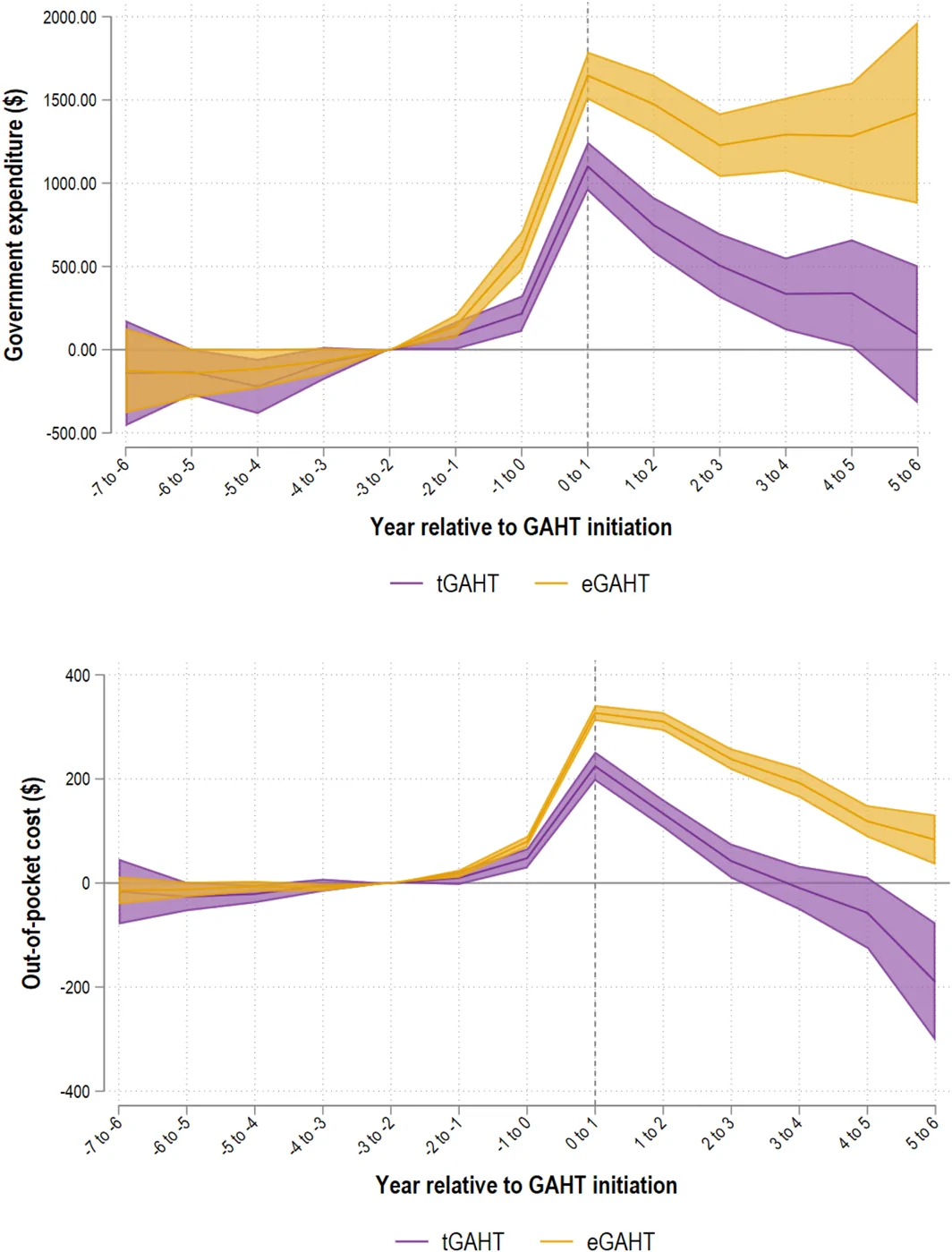
Government spending and out‐of‐pocket costs relative to GAHT initiation. Costs are total toward out‐of‐hospital services and prescription medicines. Presented in 2024 AUD. All coefficients relative to 3 years before GAHT initiation (i.e., event time = −3). GAHT = Gender‐affirming hormone therapy.

The main event‐study results for individuals initiating tGAHT and eGAHT are presented in Figure 1 (full results including pre‐initiation hypothesis testing are provided in Supporting Information S1: SM2–SM3). These figures show the changes in annual government healthcare spending and associated patient out‐of‐pocket costs per person relative to 3 years before GAHT initiation (i.e., the baseline/reference period). For both regimens, changes in costs were relatively flat prior to the reference period, with most of the point estimates overlapping zero. This provides some reassurance that prior to GAHT initiation, outcomes between treated and future treated individuals were evolving approximately in parallel over time.^6^

In the 2 years prior to GAHT initiation, there is evidence of increasing healthcare costs, aligning with increased healthcare engagement in the lead up to GAHT initiation. Total government healthcare expenditure toward out‐of‐pocket medical services and prescription medicines increased sharply in the year of GAHT initiation for both groups, peaking at AUD$1101 (95% CI 952; 1249) per person among tGAHT recipients and AUD$1647 (95% CI 1503; 1791) per person among eGAHT recipients; corresponding to a 70% and 110% increase compared to pre‐initiation levels.^7^ Expenditure then declined in subsequent years, with tGAHT recipients showing a sustained downward trajectory, falling to pre‐initiation levels by 6 years after initiation. In contrast, while eGAHT recipients also experienced reductions following the initial spike, average annual government expenditure remained above pre‐initiation levels across the follow‐up period. In the 6 years after GAHT initiation, total additional government expenditure was approximately AUD$3119 for tGAHT recipients and AUD$8348 for eGAHT recipients.

Out‐of‐pocket costs followed a similar pattern, with sharp increases observed around the year of GAHT initiation for both groups. For tGAHT recipients, out‐of‐pocket costs peaked at AUD$224 (95% CI 196; 253) per person in the initiation year—a 67% increase—before declining steadily, falling below baseline by year six. Among eGAHT recipients, out‐of‐pocket costs also rose markedly, by 113%, at initiation (peaking at AUD$327 (95% CI 311; 342) per person) and then declined thereafter. After 6 years, eGAHT recipients incurred an additional AUD$83 (95% CI 35; 131) in out‐of‐pocket costs per annum. Although they remained above pre‐initiation levels by year six, the trends suggest that increased time since initiation is associated with reduced out‐of‐pocket costs for eGAHT recipients. In the 6 years after GAHT initiation, total additional patient out‐of‐pocket costs were approximately AUD$143 for tGAHT recipients and AUD$1269 for eGAHT recipients.

### 6.2 Additional Analyses

Results showed similar dynamic patterns when estimating log‐linear models (Supporting Information S1: SM4 and SM5) and two‐part models (Supporting Information S1: SM6–SM9). For both tGAHT and eGAHT initiators, estimates conditional on positive expenditure are slightly smaller in magnitude than the baseline OLS estimates. This indicates that post‐initiation increases in costs are driven primarily by higher expenditure among individuals with positive costs (intensive margin), rather than solely by an increase in the probability of any spending (extensive margin).

Government expenditure and out‐of‐pocket cost estimates were also similar when restricting treatment and control groups to initiate within three years of each other (Supporting Information S1: SM10–SM11), with cohort confidence intervals overlapping in the year of initiation. This suggests that our cost estimates are robust to cohort composition and calendar‐time differences over the study period.

The stratification by age at GAHT initiation (15–24 and 25 years and above) for government spending and out‐of‐pocket costs are provided in Supporting Information S1: SM12 and SM13, respectively. For tGAHT initiators, the patterns of expenditure were similar for people who initiated at earlier or later years however there was some evidence that out‐of‐pocket costs were marginally higher in the year of initiation for people who initiated over 25 [AUD$295 (95% CI 235; 354)] relative to those who initiated between 15 and 24 years [AUD$174 (147; 201)]. For eGAHT initiators, government spending was slightly higher for people initiating at age 25 and above than those initiating at younger age, respectively AUD$1779 (95% CI 1542; 2015) and AUD$1460 (95% CI 1377; 1544) in the year of initiation. Out‐of‐pocket spending was however similar for both age groups.

### 6.3 Heterogeneity by Cost Categories

**FIGURE 2:**
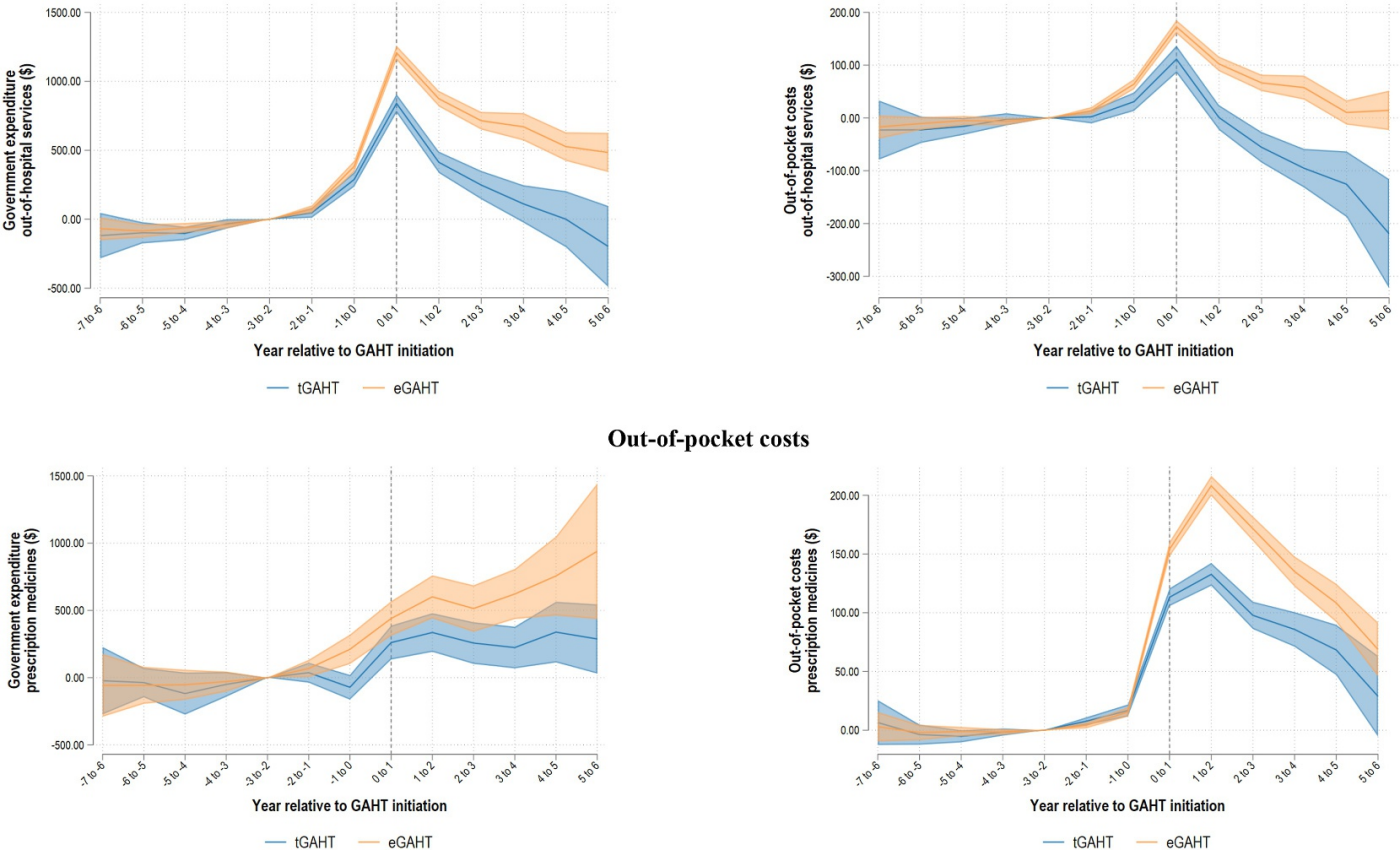
Government spending and out‐of‐pocket costs relative to GAHT initiation for out‐of‐hospital services (left) and prescription medicines (right). All coefficients relative to 3 years before GAHT initiation (i.e., event time = −3). GAHT = Gender‐affirming hormone therapy.

The costs stratified by out‐of‐hospital medical services and prescription medicines (Figure 2, full results Supporting Information S1: SM14–SM17) suggest that spikes in government expenditure around initiation are largely attributed to out‐of‐hospital services, and, for both groups, this type of expenditure reduces over time. In contrast, government expenditure toward prescription medicines steadily increases overtime, remaining higher than pre‐initiation levels across the follow‐up period. Similarly, while out‐of‐pocket costs toward out‐of‐hospital medical services fall quickly for tGAHT recipients, they remain above pre‐initiation levels for eGAHT recipients until 6 years post initiation. Out‐of‐pocket spending on prescription medicines shows a similar pattern, with persistent increases observed for both groups, although the rise is more pronounced among eGAHT recipients. These results indicate that while costs associated with out‐of‐hospital medical services diminish following initiation, higher spending on prescription medicines contributes to sustained long‐term government expenditure.

**FIGURE 3:**
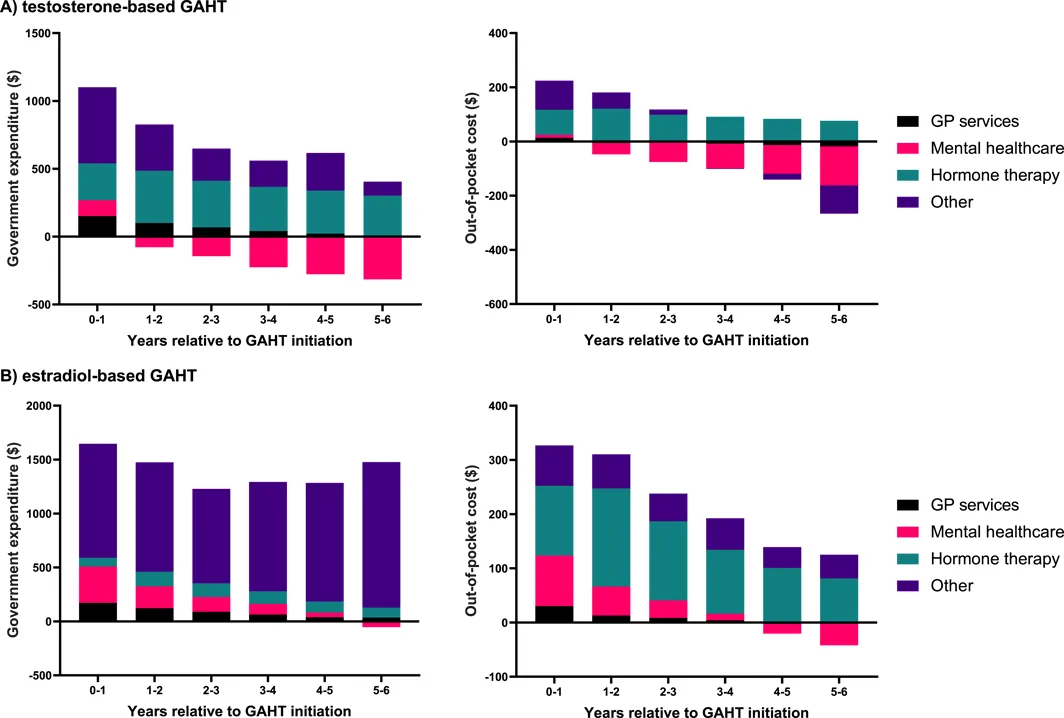
Heterogeneity in expenditure following GAHT initiation by regimen. All coefficients relative to 3 years before GAHT initiation (i.e., event time = −3). “Other” category is shown for illustrative purposes and presents the total spending less the amounts per category. Some GP services include mental health related care. GAHT = Gender‐affirming hormone therapy.

Figure 3 provides further disaggregation of government and out‐of‐pocket costs by care category. Among tGAHT recipients (Panel A), the spike in expenditure around initiation was primarily attributable to increased outlays on hormone therapy, GP services, and other out‐of‐hospital services, but these were offset in later years by substantial reductions in mental healthcare spending. By year six, the decline in government and patient mental health expenditure alone exceeded the average annual government cost of hormone therapy, indicating that savings in this domain more than covered ongoing pharmaceutical costs.

In contrast, for eGAHT recipients (Panel B), higher government expenditure and out‐of‐pocket costs persisted throughout the follow‐up period. While there were some observed reductions in mental healthcare expenditure, these were outweighed by increases in other spending (mainly non‐eGAHT prescription medication costs), resulting in total spending that remained above pre‐initiation levels across the 6 years of follow‐up. This divergence highlights regimen‐specific differences in the long‐term fiscal impact of GAHT initiation.

## 7 Discussion

In this paper, we examine healthcare costs following initiation of GAHT among 32,313 trans Australians. We found there are spikes in costs around initiation, reflecting the upfront medical engagement required to commence treatment. For tGAHT recipients, these spikes were followed by sustained declines in both government and out‐of‐pocket spending, with total expenditure returning to, or falling below baseline, after 6 years. Further, by year six, reductions in mental healthcare spending for tGAHT recipients more than offset the costs of hormone therapy. For eGAHT recipients, costs also declined after the initiation year but remained above pre‐initiation levels across the 6 years of follow‐up. Increases in prescription medicine spending for eGAHT recipients still outweighed reductions in mental healthcare costs, indicating that government cost reductions may take longer to accrue for this group.

The increased costs in the periods immediately prior to, and coinciding with, GAHT initiation, is consistent with treatment guidelines recommending concurrent monitoring, GP and specialist consultation, including mental health support (Cheung et al. 2019). The reductions in mental healthcare costs echo numerous studies documenting improvements in mental health and reductions in mental health treatment following GAHT initiation (Saxby, Buchmueller, et al. 2026; T. Campbell et al. 2023; Nolan et al. 2023) and other studies documenting lower mental healthcare utilization following gender‐affirming surgeries (Saxby and Nolan 2026; Bränström and Pachankis 2020). The comparatively smaller, and somewhat lagged, reductions in mental healthcare costs among eGAHT recipients also follows other studies which found that, compared to tGAHT, eGAHT is associated with smaller short‐term improvements in gender dysphoria and mental health outcomes (L. Foster Skewis et al. 2021). Another study in Australia also found that reductions in mental health treatment following initiation were more pronounced among eGAHT recipients than tGAHT recipients (Saxby, Buchmueller, et al. 2026). However, this study also showed that use of mental health treatment did immediately reduce among eGAHT recipients who were already engaged with care, suggesting that unmet need is also likely to play a role in these differences. Finally, we show that spending on hormone therapy is relatively stable after initiation, consistent with research on low regret and discontinuation rates following initiation of gender‐affirming medical care (I. B. Foster Skewis et al. 2026; Wiepjes et al. 2018).

The potential welfare implications associated with these costs warrants specific consideration. Recent Australian evidence from a randomized controlled trial suggests that tGAHT led to a 5.6 point reduction on the Patient Health Questionnaire‐9 scale after 3 months (Nolan et al. 2023). With a conservative estimate of a 1‐point reduction in PHQ‐9 being associated with a 0.034 QALY gain per year (Furukawa et al. 2021), this corresponds to 0.04 QALYs per year. Applying our estimated government costs (AUD$3119 per person over 6 years) yields a back‐of‐the‐envelope calculation of AUD$12,996 per QALY gained for tGAHT recipients. With a typical willingness‐to‐pay threshold of AUD$50,000 per QALY in Australia (George et al. 2001), this suggests that tGAHT is likely to be cost‐effective.

While there is less comprehensive evidence on the impacts of GAHT on QALYs for eGAHT recipients, particularly in Australia, other international studies have indicated that, following initiation, eGAHT recipients have reduced risk of moderate‐to‐severe depressive symptoms (Reisner et al. 2025; Tordoff et al. 2022) and improved overall quality of life within 1 year of initiation (Manieri et al. 2014). Using an assumed utility gain lower than our tGAHT benchmark, a conservative 0.02–0.04 QALYs per year, and our estimated government costs for eGAHT recipients (AUD$8348 per person over 6 years), yields a cost per QALY gained of $69,567–$34,783 (midpoint ≈ $52,175/QALY). Given healthcare expenditures decline in the years following initiation, should quality of life gains sustain or improve in the longer‐term, the multi‐year incremental cost‐effectiveness ratio would reduce, placing eGAHT within or near typical willingness‐to‐pay thresholds in Australia. Indeed, the observed reductions in mental healthcare spending over the 6 years following initiation supports this expectation.

Beyond these specific considerations, empirical evidence suggests that gender‐affirming care, including GAHT, can reduce mental health related hospitalisations and suicide risk among trans people (T. Campbell et al. 2023; Mann et al. 2024; Bränström and Pachankis 2020). Taken together, the evidence indicates that GAHT is likely to represent a highly cost‐effective use of public healthcare resources.

These results should be considered with the context of several limitations. First, it is important to note that this sample only comprises trans people who were able to access GAHT at some point. Many studies have noted that there are numerous barriers to accessing GAHT, including experiences of discrimination in the healthcare setting and lack of access to gender‐affirming clinics and clinicians providing GAHT (Saxby and Stephens 2024; Hafeez et al. 2017; Seelman et al. 2017). Consequently, our findings should be interpreted as the incremental costs among those actively seeking, and able to access, GAHT. However, the direction of the bias caused by this is unclear. On the one hand, individuals able to access GAHT may have higher baseline resources and better outcomes, inflating apparent benefits. On the other hand, excluding those with unmet need may underestimate the potential population‐level impact of improving access. These factors should be considered in future research.

It is also important to note that GAHT is only one aspect of gender‐affirming medical care and, due to data limitations, we cannot disentangle the effects from other types of care, such as puberty blockers or gender‐affirming surgeries. Although medical gender‐affirmation can be a non‐linear pathway, it is estimated that a large share—between 70% and 80%—have, or want to receive GAHT (K. Baker and Restar 2022; Cook et al. 2017). In a setting where costs of surgical medical care are highly prohibitive, many trans individuals seeking medical affirmation access GAHT (Bretherton et al. 2021).

As the data is currently not linked to hospital records, the costs are also likely to represent a lower bound. We cannot investigate other important costs such as mental health related emergency department visits or inpatient hospitalisations, which, in Australia, are largely borne by the government. However, given other studies have found associations with GAHT and fewer suicide attempts and mental health related hospitalizations (T. Campbell et al. 2023; Bränström and Pachankis 2020), excluding these costs likely leads us to underestimate the potential savings from reduced acute mental healthcare.

In addition, as there is currently no other information on gender identity in PLIDA, we cannot estimate changes relative to trans people who are not accessing GAHT nor understand how initiating GAHT impacts costs for nonbinary individuals. Including information on gender identity in PLIDA, such as through the Census (Saxby and Hammoud 2024), will be important to better understand the broader health and cost implications of medical gender‐affirmation.

Finally, it is important to note that, as our empirical approach relies on not yet treated individuals as controls, longer time horizons mechanically reduce the pool of not‐yet‐treated comparisons. As a result, dynamic estimates at longer horizons are identified from a narrower set of comparisons and are less precisely estimated. As data collection matures in this space, future work will allow for more robust estimation of longer‐run effects.

Despite these limitations, our study provides several unique contributions. To our knowledge, this is the largest population‐based study of GAHT recipients globally and the first to provide long‐term evidence on healthcare costs following GAHT initiation, with robust estimates by regimen. Our empirical framework also addresses biases present in studies that compare trans populations to cisgender samples, including diverging baseline health levels relative to cisgender populations (Saxby, Hutchinson‐Tovar, et al. 2025). Further, by leveraging whole‐of‐population linked administrative data with data on outcomes spanning over a decade, our study avoids the limitations associated with generalizing from small or convenience samples with short‐term follow‐up. This allows us to capture real‐world healthcare trajectories and associated costs for all GAHT recipients in Australia over longer time frames. These estimates provide a foundation for incorporating prospective evidence on long‐term quality of life measures, which can be used to enable more comprehensive cost‐effectiveness analyses in this space in the future.

These results have important implications for social and health policy. First, these estimates provide the first benchmark estimates of the healthcare costs associated with GAHT initiation in a universal healthcare setting, offering an important resource for decision makers, payers, and patient groups.

Beyond system‐level costs, the persistence of out‐of‐pocket spending among eGAHT recipients highlights the need for policies that address financial barriers directly. This need is particularly salient given that these costs are likely to accumulate alongside other aspects of gender‐affirmation that are not, or only rarely, subsidized (e.g., surgeries, speech pathology). Specifically, our results suggest that co‐payments associated with ongoing prescription medicines should be targeted.

More broadly, policy efforts could consider expanded public subsidization, targeted reimbursement, or broader efforts to reduce inequities in access to care. Of note, Australia's recently implemented a *10‐Year National Action Plan for the Health and Wellbeing of LGBTIQA + People (2025–2035)* has identified the need for safe, accessible, and inclusive healthcare as a key priority for trans people (Australian Government 2024). Given concurrent research suggests that reductions in mental health treatment are more pronounced among those already engaged in care (Saxby, Buchmueller, et al. 2026), removing structural barriers to access is essential. Eliminating state‐level restrictions on GAHT access (Queensland Government 2025), that lead to inequitable access to healthcare among younger trans individuals could help reduce both payer and patient costs in the long‐run.

These costs also need to be considered alongside ongoing human rights challenges being faced by trans people. In particular, the heightened costs among eGAHT recipients following initiation may be further exacerbated by disproportionate discrimination faced by trans women (Hughto et al. 2015). Against this backdrop, it is notable that bans on gender‐affirming medical care have at times been justified by concerns around irreversibility, regret, or poor long‐term outcomes (Noone et al. 2025). Our findings however show that on average, mental healthcare spending is lower following GAHT initiation. This suggests that gender‐affirming care may represent a more effective, and cost‐effective, long‐term treatment option for many trans people.

## 8 Conclusion

This study provides the first population‐based, long‐term evidence on healthcare costs following GAHT initiation in a universal healthcare setting. We find that GAHT initiation is associated with dynamic and heterogeneous changes in healthcare costs, peaking in the initiation year before declining substantially thereafter. Mapping these costs onto existing literature on QALY gains suggest that GAHT is cost‐effective. Altogether, findings suggest that GAHT represents a cost‐effective long‐term treatment option for many trans people, and that restricting access is likely to impose substantial health and economic costs on both patients and healthcare systems.

## Funding

This work is supported by the University of Melbourne McKenzie Fellowship (2025MCK182), a National Health and Medical Research Council Investigator Grant (2034450), and a Viertel Charitable Foundation Clinical Investigator Award.

## Ethics Statement

This study was approved by the University of Melbourne Human Research Ethics Committee (12519).

## Consent

The authors have nothing to report.

## Conflicts of Interest

The authors declare no conflicts of interest.

## Permission to Reproduce Material From Other Sources

The authors have nothing to report.

## Supporting information


Supporting Information S1

## Data Availability

Data is available upon request from the Australian Bureau of Statistics.

## Endnotes

^1^
All costs presented in 2024 dollars. 1 AUD = 0.72 USD; 0.49 GBP in 2024 using OECD purchasing power parities.

^2^
While the changes in quality of life measures for tGAHT recipients are measured from Australian data, information on other health outcomes following GAHT initiation is captured from other jurisdictions. Thus, it is important to note that the overall cost per QALY is likely measured with error. In Australia, the typical willingness‐to‐pay threshold is AUD$50,000 per QALY (USD$36,000) (George et al. 2001). Cost‐effectiveness analysis and the consistency of decision making: evidence from pharmaceutical reimbursement in Australia (1991–1996). *Pharmacoeconomics*, 19, 1103–1109.; in the United Kingdom: £20,000–£30,000 per QALY (USD$29,400–$44,100) (Claxton et al. 2015). Methods for the estimation of the National Institute for Health and Care Excellence cost‐effectiveness threshold. *Health Technology Assessment (Winchester, England)*, 19, 1.; and in the United States: USD$100,000–$150,000 (Neumann and Cohen 2017). ICER's revised value assessment framework for 2017–2019: a critique. *Pharmacoeconomics*, 35, 977–980. 1 AUD = 0.72 USD; 0.49 GBP in 2024 using OECD purchasing power parities.

^3^
Indeed, the observed reductions in mental health treatment supports that sustained or improved mental health benefits are likely.

^4^
This approach is commonly used to avoid ambiguity in eGAHT versus tGAHT assignment, particularly in cases when an individual's gender marker changes over time.

^5^
Because our empirical approach relies on staggered treatment timing and uses not‐yet‐treated individuals as controls, the number of valid comparisons mechanically declines at longer event times. This does not invalidate identification but reduces the precision of dynamic estimates at longer horizons. The 6‐year post‐initiation window was chosen to balance two considerations: (i) allowing sufficient time to observe medium‐run adjustments in healthcare utilisation and costs, and (ii) retaining an adequate pool of not‐yet‐treated comparison units.

^6^
With the exception of government expenditure towards out‐of‐hospital services, the tests of the joint null hypothesis of no differential pre‐trends (event times −7 to −3 jointly equal to zero) were not statistically significant. For the subcategory of government expenditure towards out‐of‐hospital services, there was some evidence of a modest upward pre‐initiation trend. While this suggests some deviation from strict parallel trends for this outcome, the magnitude of the pre‐period differences was modest compared to the post‐initiation changes.

^7^
These correspond to the sample mean estimated in all years prior to GAHT initiation. The results of the log‐transformed models to approximate percentage changes are also provided in Supporting Information S1: SM4‐SM5.
