# Supplementary material for: Healthcare Costs Following Medical Gender‐Affirmation: Evidence From Whole‐of‐Population Australian Administrative Data
Source: Health Econ. 2026 Jul 5;35(10):1468–81. doi: 10.1002/hec.70127 (PMC13532616; doi:10.1002/hec.70127)
Supplement: Supplementary file 1 [file HEC-35-1468-s001.docx]

# Supplementary Material

*SM. 1 – Descriptive statistics for sample at reference period (event time -3)*

|  | **Testosterone-based GAHT** | | | **Estrogen-based GAHT** | | |
| --- | --- | --- | --- | --- | --- | --- |
| **Treated and not yet treated cohorts** | **Age  GAHT start** | **Benefit paid – Out-of-hospital services and prescription medicines** | **Out-of-pocket cost – Out-of-hospital services and prescription medicines** | **Age  GAHT start** | **Benefit paid – Out-of-hospital services and prescription medicines** | **Out-of-pocket cost – Out-of-hospital services and prescription medicines** |
| Initiate 2016 | 25.61 | 1518.60 | 311.87 | 40.02 | 1847.67 | 373.81 |
| Initiate 2017-2024 | 21.59 | 1676.08 | 350.27 | 27.90 | 1362.32 | 265.90 |
| *p-value* | *<0.001* | *0.432* | *0.207* | *<0.001* | *0.025* | *<0.001* |
| Initiate 2017 | 23.93 | 1759.62 | 275.93 | 33.15 | 1529.73 | 274.93 |
| Initiate 2018-2024 | 21.45 | 1671.05 | 354.74 | 26.69 | 1324.09 | 261.66 |
| *p-value* | *<0.001* | *0.981* | *0.002* | *<0.001* | *0.211* | *0.003* |
| Initiate 2018 | 22.04 | 1440.40 | 265.14 | 33.15 | 1316.26 | 274.93 |
| Initiate 2019-2024 | 21.39 | 1694.55 | 363.87 | 26.69 | 1179.11 | 261.66 |
| *p-value* | *0.044* | *0.139* | *<0.001* | *<0.001* | *0.384* | *0.35* |
| Initiate 2019 | 21.96 | 1506.69 | 277.24 | 31.90 | 1798.98 | 267.63 |
| Initiate 2020-2024 | 21.31 | 1719.60 | 375.42 | 26.07 | 1267.09 | 260.95 |
| *p-value* | *0.029* | *0.222* | *<0.001* | *<0.001* | *0.002* | *0.615* |
| Initiate 2020 | 22.31 | 1490.46 | 311.57 | 29.95 | 1234.75 | 246.55 |
| Initiate 2021-2024 | 21.16 | 1755.07 | 385.31 | 25.45 | 1272.23 | 263.24 |
| *p-value* | *<0.001* | *0.187* | *<0.001* | *<0.001* | *0.668* | *0.19* |
| Initiate 2021 | 21.51 | 1458.90 | 332.27 | 28.23 | 1344.47 | 251.84 |
| Initiate 2022-2024 | 21.08 | 1827.41 | 398.26 | 24.76 | 1254.36 | 266.06 |
| *p-value* | *0.104* | *0.076* | *0.001* | *<0.001* | *0.62* | *0.234* |
| Initiate 2022 | 21.37 | 1779.91 | 349.36 | 25.76 | 1399.60 | 258.43 |
| Initiate 2023-2024 | 20.95 | 1847.05 | 418.48 | 24.33 | 1190.39 | 269.41 |
| *p-value* | *0.106* | *0.796* | *<0.001* | *<0.001* | *0.154* | *0.342* |
| Initiate 2023 | 20.95 | 1818.59 | 363.86 | 24.55 | 1054.82 | 247.53 |
| Initiate 2024 | 20.95 | 1874.61 | 471.38 | 24.11 | 1320.59 | 290.43 |
| *p-value* | *1* | *0.813* | *<0.001* | *0.158* | *0.026* | *0.001* |

##### SM. 2 – Event study results total government expenditure (out-of-hospital services and prescription medicines)

|  | **Testosterone-based GAHT** | | | | **Oestradiol-based GAHT** | | | |
| --- | --- | --- | --- | --- | --- | --- | --- | --- |
| **Event  time** | **Beta** | **Lower limit** | **Upper limit** | **No.**  **person-years** | **Beta** | **Lower limit** | **Upper limit** | **No.**  **person-years** |
| -7 | -141.33 | -459.53 | 176.86 | 1469 | -126.32 | -381.92 | 129.27 | 2934 |
| -6 | -134.69 | -273.41 | 4.03 | 7879 | -141.83 | -290.63 | 6.97 | 14854 |
| -5 | -220.62 | -386.21 | -55.02 | 19842 | -114.70 | -233.23 | 3.84 | 36185 |
| -4 | -82.70 | -181.61 | 16.21 | 38953 | -68.93 | -148.34 | 10.48 | 66729 |
| -3 | 0.00 | 0.00 | 0.00 | 0 | 0.00 | 0.00 | 0.00 | 0 |
| -2 | 84.33 | 0.05 | 168.61 | 50142 | 141.65 | 71.73 | 211.57 | 83948 |
| -1 | 216.60 | 107.74 | 325.45 | 37712 | 593.31 | 473.57 | 713.04 | 65268 |
| 0 | 1100.94 | 952.17 | 1249.71 | 26891 | 1646.58 | 1502.54 | 1790.63 | 48431 |
| 1 | 748.51 | 580.49 | 916.53 | 18407 | 1474.61 | 1298.43 | 1650.78 | 34117 |
| 2 | 505.33 | 311.61 | 699.04 | 12035 | 1228.10 | 1036.70 | 1419.50 | 22540 |
| 3 | 335.07 | 116.57 | 553.58 | 7018 | 1292.17 | 1070.70 | 1513.65 | 13501 |
| 4 | 339.31 | 16.05 | 662.56 | 3362 | 1282.79 | 960.86 | 1604.73 | 6932 |
| 5 | 90.17 | -324.91 | 505.26 | 1108 | 1423.58 | 876.22 | 1970.94 | 2572 |

Notes: All coefficients are relative to the reference mean at three years prior to surgery (i.e., event time=$\mathbf{-3}$). The lower and upper limits represent the bounds of the 95% confidence interval. Test of joint null hypothesis of no differential pre-trends (event times −7 to −3 are jointly equal to zero): t-GAHT p-value=0.08; e-GAHT p-value=0.24.

##### SM. 3 – Event study results total out-of-pocket costs (out-of-hospital services and prescription medicines)

|  | **Testosterone-based GAHT** | | | | **Oestradiol-based GAHT** | | | |
| --- | --- | --- | --- | --- | --- | --- | --- | --- |
| **Event  time** | **Beta** | **Lower limit** | **Upper limit** | **No.**  **person-years** | **Beta** | **Lower limit** | **Upper limit** | **No.**  **person-years** |
| -7 | -16.439 | -79.580 | 46.703 | 1469 | -14.279 | -41.137 | 12.579 | 2934 |
| -6 | -26.187 | -54.201 | 1.828 | 7879 | -12.445 | -27.207 | 2.317 | 14854 |
| -5 | -21.098 | -38.826 | -3.371 | 19842 | -6.189 | -16.531 | 4.154 | 36185 |
| -4 | -4.192 | -16.630 | 8.245 | 38953 | -8.333 | -15.778 | -0.888 | 66729 |
| -3 | 0.000 | 0.000 | 0.000 | 0 | 0.000 | 0.000 | 0.000 | 0 |
| -2 | 9.773 | -3.575 | 23.122 | 50142 | 17.920 | 10.259 | 25.581 | 83948 |
| -1 | 47.493 | 28.261 | 66.726 | 37712 | 79.591 | 68.469 | 90.713 | 65268 |
| 0 | 224.373 | 195.964 | 252.783 | 26891 | 326.743 | 311.397 | 342.090 | 48431 |
| 1 | 133.402 | 105.875 | 160.928 | 18407 | 310.343 | 292.383 | 328.303 | 34117 |
| 2 | 42.183 | 8.625 | 75.740 | 12035 | 238.005 | 217.171 | 258.838 | 22540 |
| 3 | -9.492 | -51.989 | 33.004 | 7018 | 192.504 | 163.886 | 221.121 | 13501 |
| 4 | -57.232 | -126.403 | 11.939 | 3362 | 118.667 | 87.693 | 149.640 | 6932 |
| 5 | -190.314 | -304.394 | -76.235 | 1108 | 83.083 | 34.781 | 131.385 | 2572 |

Notes: All coefficients are relative to the reference mean at three years prior to surgery (i.e., event time=$\mathbf{-3}$). The lower and upper limits represent the bounds of the 95% confidence interval. Test of joint null hypothesis of no differential pre-trends (event times −7 to −3 are jointly equal to zero): t-GAHT p-value=0.19; e-GAHT p-value=0.16).

##### SM. 4 – Event-study estimates of percentage changes in total government expenditure (log-linear specification)

|  | **Testosterone-based GAHT** | | | | **Oestradiol-based GAHT** | | | |
| --- | --- | --- | --- | --- | --- | --- | --- | --- |
| **Event  time** | **Beta** | **Lower limit** | **Upper limit** | **No.**  **person-years** | **Beta** | **Lower limit** | **Upper limit** | **No.**  **person-years** |
| -7 | -0.15 | -0.45 | 0.29 | 1469 | 0.20 | -0.16 | 0.70 | 2934 |
| -6 | -0.25 | -0.38 | -0.10 | 7879 | -0.13 | -0.27 | 0.03 | 14854 |
| -5 | -0.15 | -0.25 | -0.04 | 19842 | -0.17 | -0.26 | -0.07 | 36185 |
| -4 | -0.07 | -0.15 | 0.01 | 38953 | -0.07 | -0.15 | 0.01 | 66729 |
| -3 | 0.00 | 0.00 | 0.00 | 0 | 0.00 | 0.00 | 0.00 | 0 |
| -2 | 0.04 | -0.04 | 0.11 | 50142 | 0.16 | 0.07 | 0.25 | 83948 |
| -1 | 0.37 | 0.25 | 0.50 | 37712 | 1.25 | 1.06 | 1.47 | 65268 |
| 0 | 1.56 | 1.30 | 1.86 | 26891 | 7.17 | 6.35 | 8.08 | 48431 |
| 1 | 0.88 | 0.64 | 1.16 | 18407 | 4.41 | 3.77 | 5.14 | 34117 |
| 2 | 0.30 | 0.09 | 0.55 | 12035 | 2.36 | 1.89 | 2.91 | 22540 |
| 3 | 0.05 | -0.15 | 0.30 | 7018 | 1.17 | 0.80 | 1.62 | 13501 |
| 4 | -0.37 | -0.53 | -0.16 | 3362 | 0.47 | 0.15 | 0.88 | 6932 |
| 5 | -0.59 | -0.74 | -0.36 | 1108 | -0.18 | -0.42 | 0.16 | 2572 |

Notes: Estimates are presented as percentage changes, calculated as $\mathbf{exp}\boldsymbol{(\beta)-1}$, where β is obtained from models estimated using $\mathbf{ln}\boldsymbol{(}\text{costs}\boldsymbol{+0.01)}$. All coefficients are relative to the reference mean at three years prior to surgery (i.e., event time=$\mathbf{-3}$). The lower and upper limits represent the bounds of the 95% confidence interval.

##### SM. 5 – Event-study estimates of percentage changes in total out-of-pocket costs (log-linear specification)

|  | **Testosterone-based GAHT** | | | | **Oestradiol-based GAHT** | | | |
| --- | --- | --- | --- | --- | --- | --- | --- | --- |
| **Event  time** | **Beta** | **Lower limit** | **Upper limit** | **No.**  **person-years** | **Beta** | **Lower limit** | **Upper limit** | **No.**  **person-years** |
| -7 | -0.04 | -0.41 | 0.55 | 1469 | 0.00 | -0.31 | 0.43 | 2934 |
| -6 | -0.23 | -0.38 | -0.03 | 7879 | -0.15 | -0.30 | 0.02 | 14854 |
| -5 | -0.21 | -0.32 | -0.08 | 19842 | -0.11 | -0.22 | 0.01 | 36185 |
| -4 | -0.12 | -0.21 | -0.03 | 38953 | -0.09 | -0.17 | -0.01 | 66729 |
| -3 | 0.00 | 0.00 | 0.00 | 0 | 0.00 | 0.00 | 0.00 | 0 |
| -2 | 0.11 | 0.01 | 0.23 | 50142 | 0.21 | 0.11 | 0.32 | 83948 |
| -1 | 0.45 | 0.28 | 0.65 | 37712 | 1.27 | 1.04 | 1.52 | 65268 |
| 0 | 4.29 | 3.59 | 5.10 | 26891 | 21.15 | 18.68 | 23.93 | 48431 |
| 1 | 2.77 | 2.18 | 3.48 | 18407 | 13.60 | 11.67 | 15.82 | 34117 |
| 2 | 1.34 | 0.89 | 1.90 | 12035 | 7.75 | 6.38 | 9.37 | 22540 |
| 3 | 0.72 | 0.32 | 1.24 | 7018 | 4.57 | 3.50 | 5.88 | 13501 |
| 4 | 0.15 | -0.19 | 0.65 | 3362 | 2.88 | 1.96 | 4.09 | 6932 |
| 5 | -0.41 | -0.65 | 0.02 | 1108 | 1.57 | 0.72 | 2.83 | 2572 |

Notes: Estimates are presented as percentage changes, calculated as $\mathbf{exp}\boldsymbol{(\beta)-1}$, where β is obtained from models estimated using $\mathbf{ln}\boldsymbol{(}\text{costs}\boldsymbol{+0.01)}$. All coefficients are relative to the reference mean at three years prior to surgery (i.e., event time=$\mathbf{-3}$). The lower and upper limits represent the bounds of the 95% confidence interval.

##### SM. 6 – Event-study estimates for any government expenditure

|  | **Testosterone-based GAHT** | | | | **Oestradiol-based GAHT** | | | |
| --- | --- | --- | --- | --- | --- | --- | --- | --- |
| **Event  time** | **Beta** | **Lower limit** | **Upper limit** | **No.**  **person-years** | **Beta** | **Lower limit** | **Upper limit** | **No.**  **person-years** |
| -7 | -0.01 | -0.05 | 0.03 | 1469 | 0.03 | -0.01 | 0.06 | 2934 |
| -6 | -0.02 | -0.04 | 0.00 | 7879 | -0.01 | -0.03 | 0.01 | 14854 |
| -5 | -0.01 | -0.02 | 0.00 | 19842 | -0.01 | -0.03 | 0.00 | 36185 |
| -4 | 0.00 | -0.01 | 0.00 | 38953 | 0.00 | -0.01 | 0.00 | 66729 |
| -3 | 0.00 | 0.00 | 0.00 | 0 | 0.00 | 0.00 | 0.00 | 0 |
| -2 | 0.00 | -0.01 | 0.01 | 50142 | 0.01 | 0.00 | 0.01 | 83948 |
| -1 | 0.01 | 0.00 | 0.01 | 37712 | 0.05 | 0.04 | 0.05 | 65268 |
| 0 | 0.02 | 0.01 | 0.03 | 26891 | 0.10 | 0.09 | 0.11 | 48431 |
| 1 | 0.00 | -0.01 | 0.02 | 18407 | 0.08 | 0.07 | 0.09 | 34117 |
| 2 | -0.01 | -0.03 | 0.00 | 12035 | 0.05 | 0.04 | 0.07 | 22540 |
| 3 | -0.02 | -0.04 | 0.00 | 7018 | 0.02 | 0.01 | 0.04 | 13501 |
| 4 | -0.06 | -0.08 | -0.03 | 3362 | 0.00 | -0.02 | 0.02 | 6932 |
| 5 | -0.09 | -0.13 | -0.05 | 1108 | -0.05 | -0.08 | -0.02 | 2572 |

Notes: All coefficients are relative to the reference mean at three years prior to surgery (i.e., event time=$\mathbf{-3}$). The lower and upper limits represent the bounds of the 95% confidence interval.

##### SM. 7 – Event-study estimates for any out-of-pocket costs

|  | **Testosterone-based GAHT** | | | | **Oestradiol-based GAHT** | | | |
| --- | --- | --- | --- | --- | --- | --- | --- | --- |
| **Event  time** | **Beta** | **Lower limit** | **Upper limit** | **No.**  **person-years** | **Beta** | **Lower limit** | **Upper limit** | **No.**  **person-years** |
| -7 | -0.01 | -0.06 | 0.04 | 1469 | 0.00 | -0.04 | 0.04 | 2934 |
| -6 | -0.02 | -0.05 | 0.00 | 7879 | -0.02 | -0.04 | 0.00 | 14854 |
| -5 | -0.02 | -0.04 | 0.00 | 19842 | -0.01 | -0.03 | 0.00 | 36185 |
| -4 | -0.01 | -0.03 | 0.00 | 38953 | -0.01 | -0.02 | 0.00 | 66729 |
| -3 | 0.00 | 0.00 | 0.00 | 0 | 0.00 | 0.00 | 0.00 | 0 |
| -2 | 0.01 | 0.00 | 0.02 | 50142 | 0.02 | 0.01 | 0.03 | 83948 |
| -1 | 0.02 | 0.01 | 0.04 | 37712 | 0.07 | 0.06 | 0.08 | 65268 |
| 0 | 0.12 | 0.10 | 0.13 | 26891 | 0.24 | 0.23 | 0.26 | 48431 |
| 1 | 0.09 | 0.07 | 0.11 | 18407 | 0.20 | 0.19 | 0.22 | 34117 |
| 2 | 0.06 | 0.04 | 0.08 | 12035 | 0.16 | 0.15 | 0.18 | 22540 |
| 3 | 0.04 | 0.01 | 0.07 | 7018 | 0.13 | 0.11 | 0.15 | 13501 |
| 4 | 0.01 | -0.03 | 0.04 | 3362 | 0.11 | 0.08 | 0.14 | 6932 |
| 5 | -0.05 | -0.11 | 0.00 | 1108 | 0.07 | 0.03 | 0.11 | 2572 |

Notes: All coefficients are relative to the reference mean at three years prior to surgery (i.e., event time=$\mathbf{-3}$). The lower and upper limits represent the bounds of the 95% confidence interval.

##### SM. 8 - Event-study estimates for government expenditure conditional on spending

|  | **Testosterone-based GAHT** | | | | **Oestradiol-based GAHT** | | | |
| --- | --- | --- | --- | --- | --- | --- | --- | --- |
| **Event  time** | **Beta** | **Lower limit** | **Upper limit** | **No.**  **person-years** | **Beta** | **Lower limit** | **Upper limit** | **No.**  **person-years** |
| -7 | -165.47 | -568.13 | 237.20 | 1143 | -187.03 | -572.74 | 198.69 | 1917 |
| -6 | -157.95 | -325.53 | 9.63 | 6194 | -198.52 | -411.37 | 14.34 | 9508 |
| -5 | -270.90 | -472.82 | -68.98 | 15568 | -199.90 | -366.92 | -32.89 | 23431 |
| -4 | -82.31 | -198.77 | 34.16 | 31191 | -108.36 | -214.54 | -2.18 | 44797 |
| -3 | 0.00 | 0.00 | 0.00 | 0 | 0.00 | 0.00 | 0.00 | 0 |
| -2 | 78.31 | -16.06 | 172.68 | 42336 | 152.11 | 64.08 | 240.15 | 62074 |
| -1 | 183.06 | 58.52 | 307.60 | 31537 | 642.13 | 487.75 | 796.50 | 47394 |
| 0 | 1027.01 | 856.67 | 1197.36 | 22433 | 1577.64 | 1400.00 | 1755.28 | 35040 |
| 1 | 683.65 | 488.22 | 879.09 | 15280 | 1420.05 | 1199.17 | 1640.93 | 24557 |
| 2 | 432.77 | 207.86 | 657.68 | 10001 | 1206.62 | 966.98 | 1446.26 | 16155 |
| 3 | 247.95 | -0.12 | 496.01 | 5837 | 1312.26 | 1037.26 | 1587.26 | 9681 |
| 4 | 327.86 | -32.32 | 688.04 | 2789 | 1319.00 | 921.18 | 1716.82 | 5007 |
| 5 | 87.79 | -375.94 | 551.52 | 929 | 1542.36 | 870.51 | 2214.20 | 1954 |

Notes: All coefficients are relative to the reference mean at three years prior to surgery (i.e., event time=$\mathbf{-3}$). The lower and upper limits represent the bounds of the 95% confidence interval.

##### SM. 9 – Event-study estimates for out-of-pocket costs conditional on spending

|  | **Testosterone-based GAHT** | | | | **Oestradiol-based GAHT** | | | |
| --- | --- | --- | --- | --- | --- | --- | --- | --- |
| **Event  time** | **Beta** | **Lower limit** | **Upper limit** | **No.**  **person-years** | **Beta** | **Lower limit** | **Upper limit** | **No.**  **person-years** |
| -7 | 2.78 | -93.01 | 98.56 | 840 | -15.32 | -70.34 | 39.69 | 1228 |
| -6 | -26.77 | -70.49 | 16.96 | 4448 | -11.65 | -40.14 | 16.84 | 6064 |
| -5 | -28.29 | -56.50 | -0.08 | 11065 | -3.44 | -23.84 | 16.96 | 14919 |
| -4 | 2.31 | -16.94 | 21.56 | 22159 | -12.83 | -26.81 | 1.16 | 29540 |
| -3 | 0.00 | 0.00 | 0.00 | 0 | 0.00 | 0.00 | 0.00 | 0 |
| -2 | 4.23 | -13.63 | 22.08 | 32028 | 17.47 | 5.19 | 29.74 | 43926 |
| -1 | 32.84 | 7.29 | 58.39 | 23552 | 75.44 | 57.60 | 93.28 | 32913 |
| 0 | 166.17 | 130.60 | 201.74 | 17046 | 258.64 | 236.19 | 281.08 | 24445 |
| 1 | 74.70 | 38.62 | 110.78 | 11753 | 216.10 | 190.40 | 241.80 | 17077 |
| 2 | -20.37 | -64.47 | 23.73 | 7751 | 148.43 | 118.69 | 178.16 | 11258 |
| 3 | -82.51 | -137.58 | -27.43 | 4583 | 104.34 | 62.45 | 146.24 | 6725 |
| 4 | -136.67 | -226.74 | -46.61 | 2215 | 16.37 | -27.92 | 60.66 | 3549 |
| 5 | -264.82 | -417.24 | -112.39 | 762 | -5.30 | -75.37 | 64.76 | 1414 |

Notes: All coefficients are relative to the reference mean at three years prior to surgery (i.e., event time=$\mathbf{-3}$). The lower and upper limits represent the bounds of the 95% confidence interval.

##### SM. 10 – Event-study estimates for government expenditure by cohort of initiation for tGAHT (top) and eGAHT (bottom) initiators

##### SM. 11 – Event-study estimates for out-of-pocket costs by cohort of initiation for tGAHT (top) and eGAHT (bottom) initiators

##### SM. 12 – Event-study estimates for government expenditure by age of initiation for tGAHT (top) and eGAHT (bottom) initiators

##### SM. 13 – Event-study estimates for out-of-pocket costs by age of initiation for tGAHT (top) and eGAHT (bottom) initiators

##### SM. 14 – Event study results government expenditure out-of-hospital services

|  | **Testosterone-based GAHT** | | | | **Oestradiol-based GAHT** | | | |
| --- | --- | --- | --- | --- | --- | --- | --- | --- |
| **Event  time** | **Beta** | **Lower limit** | **Upper limit** | **No.**  **person-years** | **Beta** | **Lower limit** | **Upper limit** | **No.**  **person-years** |
| -7 | -118.81 | -283.75 | 46.12 | 1469 | -69.21 | -152.27 | 13.85 | 2934 |
| -6 | -98.16 | -175.09 | -21.23 | 7879 | -84.84 | -132.30 | -37.37 | 14854 |
| -5 | -102.52 | -151.40 | -53.63 | 19842 | -62.24 | -96.89 | -27.59 | 36185 |
| -4 | -32.94 | -66.58 | 0.69 | 38953 | -40.17 | -66.99 | -13.34 | 66729 |
| -3 | 0.00 | 0.00 | 0.00 | 0 | 0.00 | 0.00 | 0.00 | 0 |
| -2 | 47.20 | 11.16 | 83.25 | 50142 | 73.42 | 46.28 | 100.55 | 83948 |
| -1 | 288.10 | 235.93 | 340.27 | 37712 | 382.65 | 341.83 | 423.47 | 65268 |
| 0 | 840.01 | 772.96 | 907.06 | 26891 | 1206.81 | 1155.07 | 1258.54 | 48431 |
| 1 | 412.77 | 335.51 | 490.04 | 18407 | 873.54 | 817.13 | 929.95 | 34117 |
| 2 | 248.08 | 145.23 | 350.94 | 12035 | 714.27 | 650.59 | 777.96 | 22540 |
| 3 | 111.07 | -24.57 | 246.70 | 7018 | 670.30 | 570.86 | 769.75 | 13501 |
| 4 | 0.61 | -202.40 | 203.63 | 3362 | 526.79 | 423.50 | 630.07 | 6932 |
| 5 | -197.48 | -491.23 | 96.26 | 1108 | 484.05 | 342.21 | 625.89 | 2572 |

Notes: All coefficients are relative to the reference mean at three years prior to surgery (i.e., event time=$\mathbf{-3}$). The lower and upper limits represent the bounds of the 95% confidence interval. Test of joint null hypothesis of no differential pre-trends (event times −7 to −3 are jointly equal to zero): t-GAHT p-value=0.001; e-GAHT p-value<0.001).

##### SM. 15 – Event study results out-of-pocket costs out-of-hospital services

|  | **Testosterone-based GAHT** | | | | **Oestradiol-based GAHT** | | | |
| --- | --- | --- | --- | --- | --- | --- | --- | --- |
| **Event  time** | **Beta** | **Lower limit** | **Upper limit** | **No.**  **person-years** | **Beta** | **Lower limit** | **Upper limit** | **No.**  **person-years** |
| -7 | -22.96 | -79.03 | 33.11 | 1469 | -17.19 | -39.05 | 4.66 | 2934 |
| -6 | -22.43 | -47.25 | 2.40 | 7879 | -10.48 | -22.43 | 1.46 | 14854 |
| -5 | -15.92 | -31.77 | -0.07 | 19842 | -5.00 | -13.73 | 3.72 | 36185 |
| -4 | -2.63 | -14.09 | 8.82 | 38953 | -6.89 | -13.53 | -0.25 | 66729 |
| -3 | 0.00 | 0.00 | 0.00 | 0 | 0.00 | 0.00 | 0.00 | 0 |
| -2 | 2.14 | -10.31 | 14.59 | 50142 | 13.83 | 7.05 | 20.60 | 83948 |
| -1 | 30.76 | 13.23 | 48.29 | 37712 | 64.03 | 54.39 | 73.66 | 65268 |
| 0 | 111.15 | 85.46 | 136.85 | 26891 | 172.61 | 160.01 | 185.21 | 48431 |
| 1 | 0.71 | -22.89 | 24.30 | 18407 | 102.28 | 88.50 | 116.06 | 34117 |
| 2 | -55.58 | -84.61 | -26.55 | 12035 | 66.43 | 50.98 | 81.87 | 22540 |
| 3 | -95.27 | -131.92 | -58.62 | 7018 | 57.48 | 34.69 | 80.28 | 13501 |
| 4 | -125.55 | -187.74 | -63.36 | 3362 | 10.34 | -12.59 | 33.26 | 6932 |
| 5 | -219.17 | -322.49 | -115.85 | 1108 | 14.33 | -22.96 | 51.61 | 2572 |

Notes: All coefficients are relative to the reference mean at three years prior to surgery (i.e., event time=$\mathbf{-3}$). The lower and upper limits represent the bounds of the 95% confidence interval. Test of joint null hypothesis of no differential pre-trends (event times −7 to −3 are jointly equal to zero): t-GAHT p-value= 0.30; e-GAHT p-value=0.15.

##### SM. 16 – Event study results government expenditure prescription medicines

|  | **Testosterone-based GAHT** | | | | **Oestradiol-based GAHT** | | | |
| --- | --- | --- | --- | --- | --- | --- | --- | --- |
| **Event  time** | **Beta** | **Lower limit** | **Upper limit** | **No.**  **person-years** | **Beta** | **Lower limit** | **Upper limit** | **No.**  **person-years** |
| -7 | -22.52 | -273.21 | 228.17 | 1469 | -57.11 | -290.65 | 176.42 | 2934 |
| -6 | -36.53 | -145.57 | 72.51 | 7879 | -56.99 | -194.39 | 80.41 | 14854 |
| -5 | -118.10 | -274.61 | 38.41 | 19842 | -52.46 | -163.89 | 58.97 | 36185 |
| -4 | -49.76 | -141.62 | 42.11 | 38953 | -28.76 | -102.84 | 45.32 | 66729 |
| -3 | 0.00 | 0.00 | 0.00 | 0 | 0.00 | 0.00 | 0.00 | 0 |
| -2 | 37.12 | -37.10 | 111.34 | 50142 | 68.23 | 4.43 | 132.03 | 83948 |
| -1 | -71.50 | -163.26 | 20.26 | 37712 | 210.66 | 101.32 | 319.99 | 65268 |
| 0 | 260.93 | 134.82 | 387.03 | 26891 | 439.78 | 311.72 | 567.83 | 48431 |
| 1 | 335.74 | 192.26 | 479.22 | 18407 | 601.06 | 441.46 | 760.66 | 34117 |
| 2 | 257.24 | 102.61 | 411.87 | 12035 | 513.83 | 341.43 | 686.23 | 22540 |
| 3 | 224.01 | 69.36 | 378.65 | 7018 | 621.87 | 435.88 | 807.86 | 13501 |
| 4 | 338.69 | 113.53 | 563.85 | 3362 | 756.00 | 462.67 | 1049.34 | 6932 |
| 5 | 287.66 | 30.54 | 544.77 | 1108 | 939.53 | 435.47 | 1443.60 | 2572 |

Notes: All coefficients are relative to the reference mean at three years prior to surgery (i.e., event time=$\mathbf{-3}$). The lower and upper limits represent the bounds of the 95% confidence interval. Test of joint null hypothesis of no differential pre-trends (event times −7 to −3 are jointly equal to zero): t-GAHT p-value=0.67; e-GAHT p-value=0.89.

##### SM. 17 – Event study results out-of-pocket costs prescription medicines

|  | **Testosterone-based GAHT** | | | | **Oestradiol-based GAHT** | | | |
| --- | --- | --- | --- | --- | --- | --- | --- | --- |
| **Event  time** | **Beta** | **Lower limit** | **Upper limit** | **No.**  **person-years** | **Beta** | **Lower limit** | **Upper limit** | **No.**  **person-years** |
| -7 | 6.52 | -12.57 | 25.60 | 1469 | 2.92 | -9.63 | 15.46 | 2934 |
| -6 | -3.76 | -12.35 | 4.83 | 7879 | -1.96 | -8.43 | 4.51 | 14854 |
| -5 | -5.18 | -10.37 | 0.02 | 19842 | -1.19 | -5.11 | 2.73 | 36185 |
| -4 | -1.56 | -4.60 | 1.49 | 38953 | -1.44 | -3.81 | 0.93 | 66729 |
| -3 | 0.00 | 0.00 | 0.00 | 0 | 0.00 | 0.00 | 0.00 | 0 |
| -2 | 7.63 | 4.35 | 10.91 | 50142 | 4.09 | 1.60 | 6.58 | 83948 |
| -1 | 16.73 | 11.60 | 21.86 | 37712 | 15.57 | 11.69 | 19.44 | 65268 |
| 0 | 113.22 | 105.69 | 120.75 | 26891 | 154.14 | 147.93 | 160.34 | 48431 |
| 1 | 132.70 | 123.01 | 142.38 | 18407 | 208.06 | 199.59 | 216.54 | 34117 |
| 2 | 97.76 | 86.13 | 109.40 | 12035 | 171.58 | 161.18 | 181.98 | 22540 |
| 3 | 85.78 | 71.03 | 100.52 | 7018 | 135.02 | 122.13 | 147.91 | 13501 |
| 4 | 68.32 | 46.91 | 89.73 | 3362 | 108.33 | 92.02 | 124.64 | 6932 |
| 5 | 28.86 | -5.49 | 63.21 | 1108 | 68.76 | 45.80 | 91.71 | 2572 |

Notes: All coefficients are relative to the reference mean at three years prior to surgery (i.e., event time=$\mathbf{-3}$). The lower and upper limits represent the bounds of the 95% confidence interval. Test of joint null hypothesis of no differential pre-trends (event times −7 to −3 are jointly equal to zero): t-GAHT p-value=0.32; e-GAHT p-value=0.68.
